# Type 2 Diabetes Mellitus as a Risk Factor for Alzheimer’s Disease: Review and Meta-Analysis

**DOI:** 10.3390/biomedicines10040778

**Published:** 2022-03-27

**Authors:** Athanasia Athanasaki, Konstantinos Melanis, Ioanna Tsantzali, Maria Ioanna Stefanou, Sofia Ntymenou, Sotirios G. Paraskevas, Theodosis Kalamatianos, Eleni Boutati, Vaia Lambadiari, Konstantinos I. Voumvourakis, George Stranjalis, Sotirios Giannopoulos, Georgios Tsivgoulis, George P. Paraskevas

**Affiliations:** 1 Department of Neurology, Evangelismos Hospital, 10676 Athens, Greece; athanasia.athan@yahoo.gr (A.A.); sofia_ntymenou@hotmail.com (S.N.); 2 2nd Department of Neurology, School of Medicine, National and Kapodistrian University of Athens, “Attikon” University General Hospital, 12462 Athens, Greece; melaniskos@gmail.com (K.M.); docjo1989@gmail.com (I.T.); marianna421@hotmail.co.uk (M.I.S.); sotirispar5@gmail.com (S.G.P.); cvoumvou@otenet.gr (K.I.V.); ssgiannopoulos@gmail.com (S.G.); tsivgoulisgiorg@yahoo.gr (G.T.); 3 1st Department of Neurosurgery, School of Medicine, National and Kapodistrian University of Athens, Evangelismos Hospital, 10676 Athens, Greece; tkalamatian@med.uoa.gr (T.K.); stranjal@otenet.gr (G.S.); 4 2nd Department of Internal Medicine, School of Medicine, National and Kapodistrian University of Athens, “Attikon” University General Hospital, 12462 Athens, Greece; boutati@otenet.gr (E.B.); lambadiarihbc@gmail.com (V.L.)

**Keywords:** type 2 diabetes mellitus, Alzheimer’s disease, common mechanisms

## Abstract

Alzheimer’s disease is the most common type of dementia, reaching 60–80% of case totals, and is one of the major global causes of the elderly population’s decline in functionality concerning daily life activities. Epidemiological research has already indicated that, in addition to several others metabolic factors, diabetes mellitus type 2 is a risk factor of Alzheimer’s disease. Many molecular pathways have been described, and at the same time, there are clues that suggest the connection between type 2 diabetes mellitus and Alzheimer’s disease, through specific genes, autophagy, and even inflammatory pathways. A systematic review with meta-analysis was conducted, and its main goal was to reveal the multilevel connection between these diseases.

## 1. Introduction

Alzheimer’s disease is the most common type of dementia. In 2020, as many as 5.8 million Americans were living with AD, and this number is projected to nearly triple to 14 million people by 2060 [1]. Approximately 95% of AD patients are over 65 years old, and they form the late-onset group of the total AD population. The remaining 5%—familial AD (fAD)—consists of patients who manifest the disease in their thirties, forties or fifties and carry genetic mutations in three genes (as currently known): amyloid precursor protein (APP), presenilin-1 (PS-1) and presenilin-2 (PS-2). All three of these genes follow autosomal dominant inheritance, their mutations’ penetrance is nearly 100%, and they are involved in the molecular processing of b-amyloid. APP is the responsible gene for the precursor protein of beta-amyloid encoding, whereas PS-1 and PS-2 code important components of the γ-secretase, a proteolytic enzyme required for the generation of b-amyloid from APP.

The etiology of late-onset AD is more complex and encompasses genetics, the strongest one being the epsilon four allele of the apolipoprotein E (ApoE4) gene, as well as other factors such as obesity, diabetes, depression and hypertension [2,3]. The APOE gene, on chromosome 19, encodes the brain’s major cholesterol transporter and occurs in three allele variants: ε2 (less common frequency in the population), ε3 (most common) and ε4. Each person has two alleles, and the presence of one or two ε4 alleles increases the risk of AD and reduces the average age of symptom onset. However, the ε2 allele is protective for AD. Major studies have emphasized APOEε4 significance in cognitive impairment, with an autopsy study [4] that focused on the interactions of DM, the subsequent high risk of cerebral amyloid angiopathy (CAA) and the formation of neurofibrillary tangles and amyloid plaques [4,5].

In this context, earlier studies have focused on the incidence rate of diabetes in AD patients [6,7,8]. One such example, the Rotterdam study, a large prospective study published in 1999, revealed an increased relative risk of diabetics, especially for those on insulin treatment for AD [6]. In the same context, intranasal insulin has been previously indicated as a potential therapeutic agent for amnestic cognitive impairment [9,10,11].

Diabetes mellitus (DM) is a chronic metabolic disorder, whose prevalence increases in elderly people [12] and as a major midlife vascular risk factor, it has been studied regarding amyloid deposition in the human brain [13]. This neuropathologic approach is required mostly in the definition of AD, according to the new National Institute on Aging and Alzheimer’s Association (NIA-AA) research framework (2018) [14], which impresses upon a biologically based definition, emphasizing the importance of biomarkers.

In order to confirm T2DM as a risk factor of AD, we conducted a quantitative meta-analysis of longitudinal studies, and we summarized the main common molecular mechanisms.

## 2. Materials and Methods

**Figure 1:**
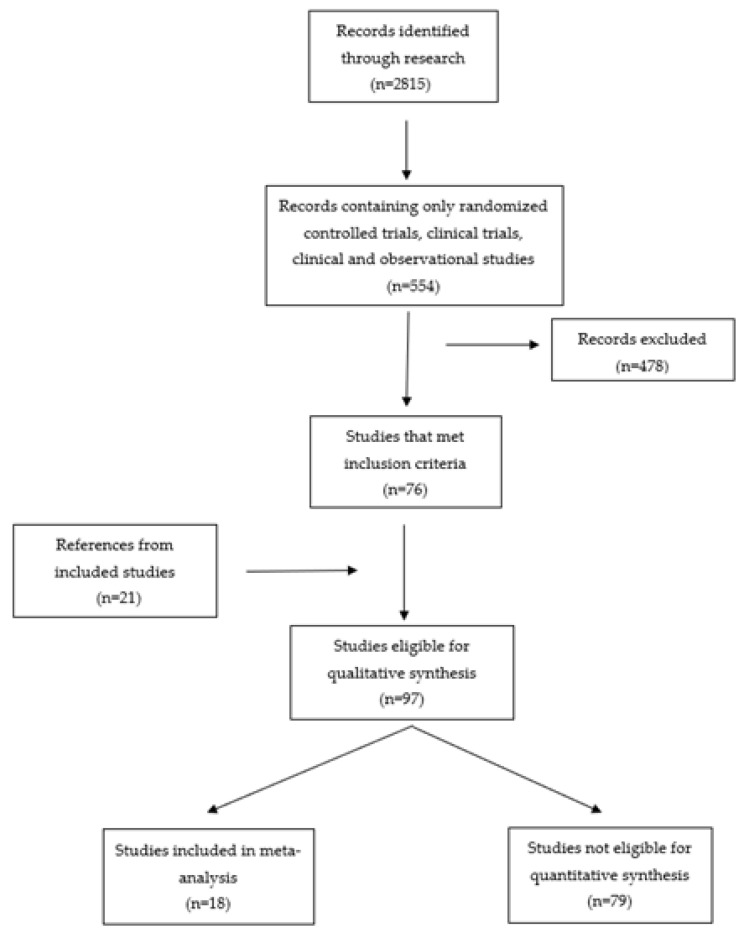
PRISMA flow diagram for the selection of included studies.

This study was based on the PRISMA statement (Figure 1). The electronic database of PubMed was searched from January 2008 to May 2021. The search terms were “type 2 diabetes mellitus” OR “diabetes” OR “vascular risk factor” OR “metabolic syndrome” AND “dementia” OR “Alzheimer disease” OR “p-tau” OR “b-amyloid”. There were 12,815 results, but by focusing on randomized controlled and clinical trials, as well as clinical and observational studies, there were 554 results. Initially, all the articles were screened according to their title and abstract, and some of them, which were not potentially relevant, were excluded (478). Additional relevant articles and reviews were retrieved for methodological issues and underlying mechanisms that were eligible for our qualitative synthesis (97).

### Inclusion/Exclusion Criteria

For meta-analytic purposes, we included only human studies that contained mainly patients with diabetes mellitus. The studies reported data concerning odds ratios (OR), risk ratios (RR) or hazard ratios (HR), and dementia was defined according to the generally accepted criteria, mostly the NINCDS-ADRDA criteria. In respect to diabetes, there was variation in the parameters, which articles used in order to define it. More specifically, most of the medical history information, including DM, was based on national health insurance records, and some articles took for granted the presence of DM without a well-defined parameter. Nevertheless, there were studies that used the fasting plasma glucose (≥126 mg/dL) or the 75 g oral glucose tolerance test or the variations of fasting plasma glucose and glycated hemoglobin (HbA1c) represented by the coefficient of variation (CV). The duration of DM was also counted in one study, and a statistical significance was noticed over five years. Furthermore, studies with dementia subjects at baseline were also included. In addition, only papers published in English or translated into English were included. We excluded animal studies, case reports and reviews, for quantitative synthesis.

## 3. Statistical Analysis

The meta-analysis was performed following the random effects model with DerSimonian Laird method, as performed by the RevMan software. Although T^2^ and X^2^ were also reported, I^2^ was the most important measure of heterogeneity (low < 25%, moderate 25–75%, high >75%). For publication bias, a funnel plot was created (Trim and Fill method), and Egger’s regression was performed using Meta-Essentials 1.5 (2020) [15]. Different meta-analyses were performed for publications reporting odds ratios, risk ratios or hazard ratios. Subgroup analyses were also performed.

## 4. Results

### Meta-Analysis

**Figure 2:**
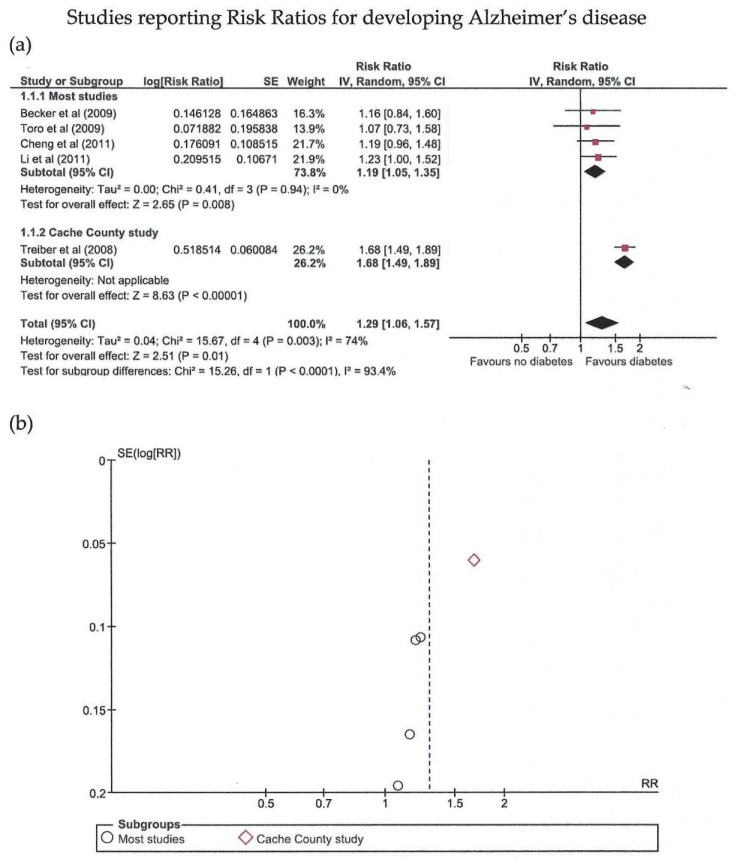
Meta-analysis (using the random effects model with the DerSimonian-Laird method) of studies reporting relative risks (RR) [17,19,20,23,33]. (**a**) Forest plot: effect sizes are represented as red squares with 95% confidence intervals. In the summary rows, the weighted average effect (or “combined” effect size) is represented as a diamond. (**b**) Funnel plot of the five studies included.

**Figure 3:**
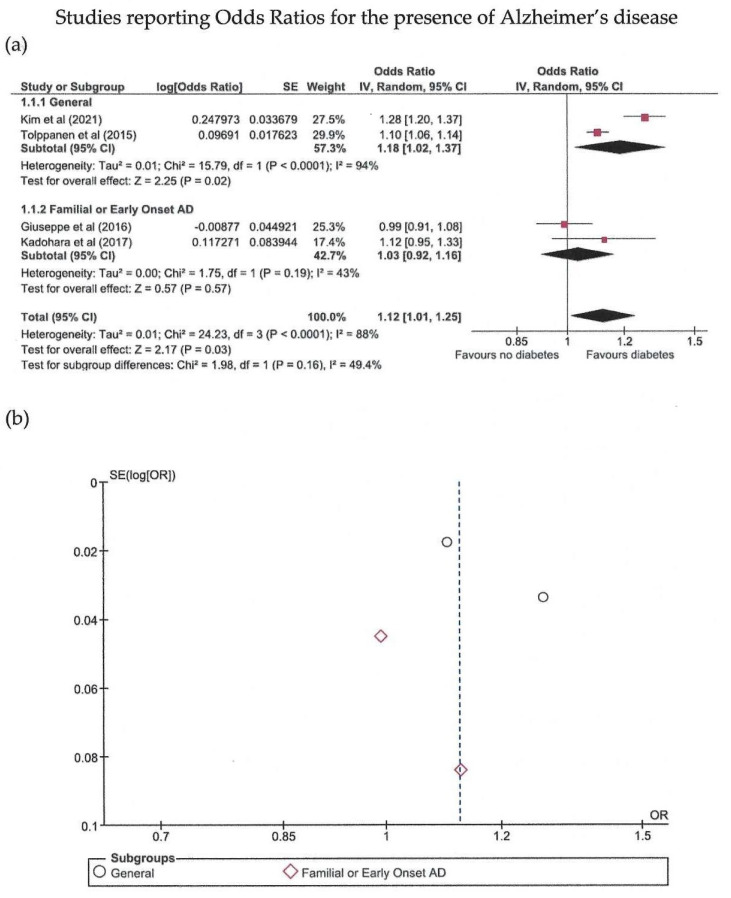
Meta-analysis (using the random effects model with the DerSimonian-Laird method) of studies reporting odds ratios (OR) [26,27,28,31]. (**a**) Forest plot: effect sizes are represented as red squares with 95% confidence intervals. In the summary rows, the weighted average effect (or “combined” effect size) is represented as a diamond. (**b**) Funnel plot of the four studies included.

**Figure 4:**
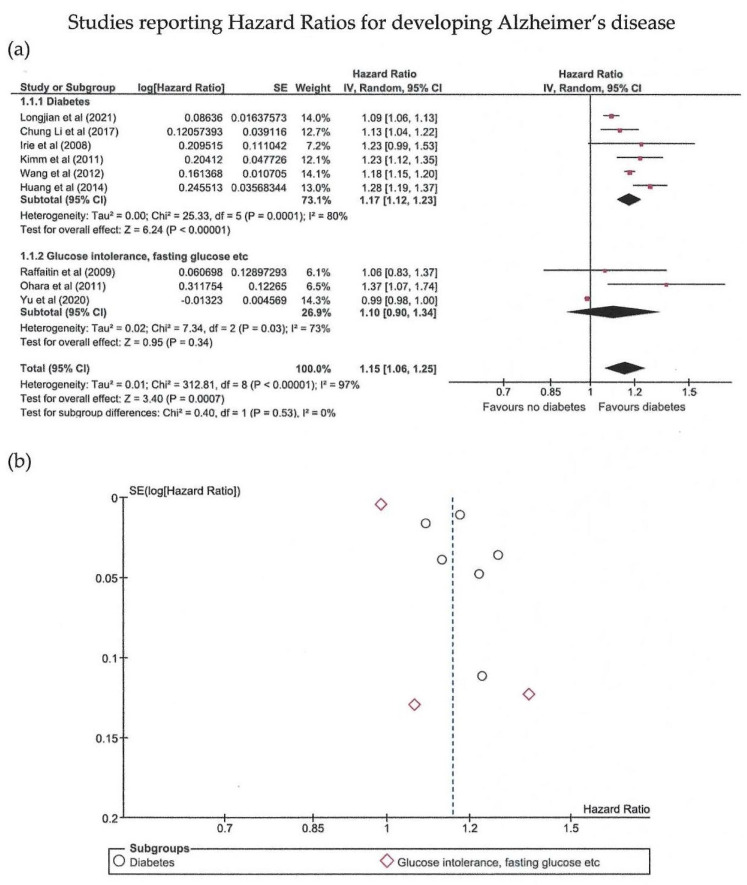
Meta-analysis (using the random effects model with the DerSimonian-Laird method) of studies reporting hazard ratios (HR) [16,18,21,22,24,25,29,30,32]. (**a**) Forest plot: effect sizes are represented as red squares with 95% confidence intervals. In the summary rows, the weighted average effect (or “combined” effect size) is represented as a diamond. (**b**) Funnel plot of the nine studies included.

Eighteen studies were included in the meta-analysis (Table 1) [16,17,18,19,20,21,22,23,24,25,26,27,28,29,30,31,32,33]. The meta-analysis was conducted separately regarding the measure of probability used; thus, there are three main groups (Figure 2, Figure 3 and Figure 4).

Risk ratios were reported by five studies with a total of 6628 patients, showing significantly increased risk ratios for AD in patients with abnormal glucose metabolism (*p* = 0.01) and moderate heterogeneity as expressed by an I^2^ of 74%. Egger’s test revealed no significant publication bias (*p* = 0.797). When removing the (statistically significant) Cache County study, the other studies showed significantly increased risk ratios for AD in patients with diabetes (*p* = 0.008), with no heterogeneity.

Odds ratios were reported by four studies, with a total of 120,645 patients showing significantly increased odds ratio for AD in patients with abnormal glucose metabolism (*p* = 0.03), yet with high heterogeneity. Egger’s test did not reveal significant publication bias (*p* = 0.744). Two of the studies were performed in the general populations and another two in subjects with early onset and/or familial AD. Only in the former was diabetes significantly associated with AD (*p* = 0.02), with high heterogeneity.

Hazard ratios were reported by nine studies, with a total of 4,229,828 patients showing significantly increased hazard ratios for AD in patients with abnormal glucose metabolism (*p* = 0.0007). High heterogeneity was observed with an I^2^ of 97%. Egger’s test revealed that publication bias was only marginally significant (*p* = 0.053). Subgroups with either diabetes or other definitions of abnormal glucose metabolism (glucose intolerance, fasting glucose levels) showed significant results, but heterogeneity was again moderate at best.

## 5. Discussion

### 5.1. Meta-Analyses

The present findings suggest that an abnormal glucose metabolism in general may be associated with an increased risk for developing Alzheimer’s disease in the general population. Publication bias may not be significant. However, heterogeneities are generally moderate or high, probably as a result of different definitions of abnormal glucose metabolism, including diabetes, fasting plasma glucose levels, Hb1Ac levels and glucose intolerance as tested by the oral glucose tolerance test, commonly preferred for the characterization of prediabetes. Different study designs may also contribute to the heterogeneity, as was shown with the study (Treiber et al. [33]) of a subsample of the Cache County Study, which was cross-sectional with retrospective examination of vascular conditions in relation with probable or possible AD with neuropsychiatric symptoms. In addition, the effect of diabetes may not be significant in early-onset or familial AD, where specific genetic factors may be responsible.

### 5.2. The Role of Insulin Resistance, Hyperinsulinemia and Hyperglycemia in AD Pathology

As already mentioned, numerous lines of evidence indicate the etiological correlation between AD, DM, and diabetes mellitus types 2 in particular because of its higher prevalence. However, type 1 DM-related defects in cognition have been examined, especially from certain perspectives such as the frequency of diabetic ketoacidosis [34].

The cornerstone of the aforementioned correlation is hyperglycemia, insulin resistance and hyperinsulinemia, which are the main features of T2DM [35].

Insulin is an anabolic hormone that promotes glucose uptake, glycogenesis, lipogenesis and protein synthesis of skeletal muscle, and it is released from the pancreas. It is transported to the brain through a receptor-mediated process [36] and accomplishes its function through the tyrosine kinase receptor pathway. Insulin receptors are abundantly expressed in the hypothalamus, cortex, hippocampus and the olfactory bulb [37], and they exist in two isoforms (IR-A and IR-B), with IR-A as the one that is mainly located in the adult nervous system [38].

IR expression in the aforementioned sites indicates important roles in synaptic plasticity, memory and homeostatic regulation [39]. This latter function, most prominent in peripheral tissues, is carried out principally by GLUT4 and GLUT8 transporters, the known insulin responsive transporters [40] that eventually translocate to the plasma membrane. There are also other glucose transporters, including GLUT1 and GLUT3, that are independent of insulin levels, are expressed at the blood brain barrier (BBB) and are responsible for neuron glucose uptake from blood [40].

In a healthy state, insulin binds to the IR, it is auto-phosphorylated because its four subunits interact, and in turn, it activates insulin-receptor substrates. There are six identified protein substrates that constitute the IRS family, and IRS-1 and -2 are those that mediate downstream pathways in the brain. Thus, IRS initiates a signaling cascade through phosphatidylinositol-3-kinase (PI3K), which activates protein kinase B (PKB or AKT). The latter one phosphorylates glycogen synthase kinase 3beta (GSK-3β), resulting in its inhibition [41]. When diabetes mellitus type 2 impacts insulin resistance, at first, the pancreas releases increased levels of insulin in order to compensate. However, chronically, insulin receptors at the BBB are downregulated, and the amount of insulin that enters the brain decreases [42].This phenomenon is called brain insulin resistance and gives prominence to the key role of GSK-3β in AD pathogenesis. In particular, GSK-3β is hyperactive and can phosphorylate tau, which aggregates to form neurofibrillary tangles [43].

In light of the hallmarks of AD pathology, which are intracellular neurofibrillary tangles and extracellular Ab plaques, it is important to emphasize that hyperinsulinemia contributes to Ab toxicity as well. IDE is the metalloprotease insulin-degrading enzyme, responsible for the degradation of both Ab and insulin [44]. In T2DM, peripheral hyperinsulinemia causes Ab accumulation as a result of competition for IDE [45]. There is also evidence that brain insulin resistance may upregulate APP and b-secretase 1 (BACE 1) and increase, by extension, Ab formation [46].

At that point, the involvement of another crucial factor is necessary, as it can prevent the extent of the aforementioned processes. This factor is Glucagon-like peptide 1 (GLP-1), which is a product of post-translational processing on the proglucagon gene and is synthesized, including in the gastrointestinal tract, in the brainstem from the neurons of nucleus tractus solitarius, and it is transmitted to several cortical and subcortical areas, specifically to the hippocampus and cortical pyramidal neurons, of which those sites are abundant in GLP-1 receptors. GLP-1 mediates insulin secretion, decreases glucagon production and has protective properties on pancreatic βcells [47]. The regulation of peripheral insulin release prevents the development of hyperinsulinemia, which is associated with brain insulin resistance and reduced signaling. Thus, GLP-1 acts favorably on neuronal plasticity and prevents neurons from apoptosis [48,49].

In addition, hyperglycemia plays a significant role in endothelial damage through the formation of advanced glycation end products (AGEs). These are glycated proteins and lipids formed by non-enzymatic glycosylation. The accumulation of AGEs provokes the subsequent increased expression of receptors of AGEs (RAGE) [50] which in turn stimulates inflammation, vascular injury [51] and oxidative stress. Mitochondrial dysfunction seems to be fundamental in AD pathogenesis [52]. Many research studies have focused on this theory and have included calcium imbalance in the common pathophysiological pathways of these two major diseases on a molecular level [53]. In terms of oxidative stress, a dysfunction in calcium homeostasis can activate abnormal calcium- and calmodulin-dependent protein kinases, with a final adverse effect in neuronal synapses [54].

### 5.3. The Role of Inflammatory Pathways

Inflammatory processes play a crucial role in the pathophysiological pathways linking AD and T2DM [55,56]. In T_2_DM, as well as in insulin resistance, there is increased production of IL-6, IL-1β, IL-18, tumor necrosis factor (TNF-a), alpha-1 antichymotrypsin and C-reactive protein [57,58,59]. The BBB in DM exhibits increased permeability [60] and is more susceptible to penetration from cytokines produced from the periphery, as it was noticed in a transgenic mouse model of AD [61]. Thus, the nervous tissue of AD patients may be more susceptible to peripheral inflammatory processes [62].

Microglia express various receptors such as CD_14_, CD_36_, CD_47_, a_6_b_1_ integrin, RAGE and Toll-like receptors which interact with Aβ oligomers [63]. In vitro findings indicate that the direction of microglia against Aβ can be regulated by the interaction of RAGE to Aβ [64]. Moreover, the connection of Aβ with CD_36_ or TLR_4_ can mobilize several inflammatory processes that augment neuronal destruction in specific areas of the brain in AD [63]. In T_2_DM, insulin resistance produces AGEs due to oxidative stress, which leads to glucotoxicity and disruption of insulin signaling [65]. In various neurodegenerative diseases such as AD, these molecules interact with Toll-like receptors (TLRs) and RAGEs [65]. More specifically, in AD, interaction of RAGE with Aβ plays a crucial role in the pathophysiology of the disease.

The JNK (c-Jun N-terminal kinase) pathway is also important in insulin resistance and is a part of the signaling cascade of tumor necrosis factor a (TNF-a) [66]. This pathway is closely related to other stress kinases such as IKK (inhibitory kb kinase) and PKR (double stranded RNA-dependent protein kinase) [67]. The IRS-1 protein is phosphorylated by the JNK and Ikba in the inhibitory serine 307 residue, resulting in a reduced effect of insulin attachment [68,69]. In AD patients, TNF-a, which is mainly produced by microglial cells, is augmented [70]. In this neurodegenerative disease, Aβ O (Aβ oligomers) aggregation results in the disposal of insulin receptors from the surface of cells [71,72,73]. The accumulation of Aβ in the brain causes the increased production of TNF-a from microglia, while Aβ oligomers trigger their receptors in neurons and stimulate stress kinases (JNK, PKR, IKK) additionally to TNF-a action [74,75,76]. IKK and PKR promote restriction of IRS-1 through Aβ oligomers in neurons of the hippocampus [74,76].

NFkB (nuclear factor kappa-light-chain-enhancer of activated B cells), which is provoked by a state of increased glucose and neuronal destruction, plays a significant role in the inflammatory process induced by diabetes [77,78]. Its activation causes the production of multiple inflammatory cytokines such as TNF-a and IL-6 [79] and can induce apoptosis. It has been suggested that, in streptozotocin-injected rats (a well-studied model mimicking DM), activation of NFkB increases reactive oxygen species (ROS) in their hippocampus [68,80].

### 5.4. The Role of Autophagy

Autophagy is a degradative process that is used by cells to eliminate defective parts via a lysosome-dependent pathway [81]. Different forms of autophagy have been classified: macroautophagy, microautophagy, chaperone-mediated autophagy (CMA) and crinephagy [82]. One of the most common types of autophagy in neurons is macroautophagy, and it is severely impacted in AD patients [83,84]. Beclin 1 is a protein taking part in autophagy, and when it is expressed deficiently in neuronal cultures of transgenic mice, it can lead to increased production of Aβ and amyloid plaques [85]. Moreover, the absence of ATG7 (autophagy-related protein 7), another autophagy protein, in the forebrain of KO (knockout) mice, can lead to increased concentrations of phospho-tau in the neurons [86]. However, autophagic processes are also affected in T2DM patients’ brain, since insulin resistance leads to increased generation of ROS, which impairs the ER (endoplasmic reticulum) and mitochondria, resulting in increased aggregation of misfolded proteins [87,88]. In order to investigate the connection between AD and DM as far as the autophagic processes are concerned, experimental models (OLEF rats) have been used [88]. In OLEF rats, increased concentrations of tau and phosphor-tau were combined with the reduction of p62 protein, which is crucial for the autophagic degradation of tau [88]. In the brains of AD and T2DM mice models, a decrease in the levels of ATG7 and LC3-II (microtubule-associated proteins 1A/1B light chain 3B) has been observed, which may be important in autophagic pathways [89]. In conclusion, in T2DM, there is augmented oxidative stress that can cause dysfunction in autophagy, contributing to the gradual aggregation of proteins such as Aβ and tau, which can lead to the appearance of AD [35].

### 5.5. Ab Oligomers and Amylin

The significance of Aβ oligomers in synaptic toxicity and their connection with brain insulin resistance has been described [90]. Aβ oligomers can bind to various receptors, such as RAGE and NMDA receptors, enhancing LTD (long term depression) at the synapses [91]. It is known that while LTP (long-term potentiation) promotes memory and is mediated by the GSK-3β pathway [92], LTD contributes to the generation and progression of dementia [93].

Of note, recent studies have focused on another amyloidogenic molecule, namely amylin (islet amyloid polypeptide, IAPP), a pancreatic hormone that is oversecreted in insulin resistance [94] and can accumulate in brain tissue as well as in cerebral vessels [95]. This research provides an additional metabolic link between T2DM and AD since it indicates Aβ–amylin interaction and the presence of aggregated amylin in patients with both these diseases [96].

## 6. Concluding Remarks

Numerous studies have previously investigated the putative association between AD and other vascular risk factors, which could be modifiable, as in dyslipidemia [97], and is especially dependent upon insulin resistance [98]. The link with obesity through the excessive amount of free fatty acids (FFAs) and changes in the macrophages’ phenotype within adipose tissue has also been indicated [99]. All these parameters pinpoint to the metabolic risk of AD and raise concepts about possible therapeutic implications. Considerable evidence highlights the potential benefit of antidiabetic drugs in AD [100], with many positive results indicated following the use of metformin, PPAR-γ and GLP-1 agonists.

The hypoglycemic and neuroprotective properties of GLP-1 have led to the production of a variety of agonists. Many of those have been established in everyday practice as important anti-diabetic agents. In AD mouse models, these agents have shown the potential to suppress microglial activation and Aβ accumulation, thus delaying the disease course and ameliorating cognitive performance [101,102]. Liraglutide has been investigated in animal experimental models, as well as in randomized controlled trials, with promising results, showing increases in brain insulin signaling, and a possible effect on mental scale scores of patients with mild cognitive impairment [102,103,104]. Exenatide is another agent in the spotlight that has shown not only neuroprotective properties in AD transgenic mice [105,106], but also effects in mouse models of Parkinson’s disease. Thus, mice treated with exendin-4 showed an increase in dopamine levels and in the density of neurons in the substantia nigra [107,108]. The GLP-1 analogue semaglutide may also protect from Aβ toxicity [109], and a clinical trial (EVOKE) has been launched to test its effect on early-onset AD.

An important observation concerns the impact of sex on AD incidence, indicated by previous studies on populations, such as postmenopausal women [32]. There is evidence that older women have an augmented incidence rate of AD dementia [110], and this phenomenon could be the result of an inflammatory imbalance, which is more prominent in females, especially in older ones due to the reduction of sex steroid hormones (e.g., progesterone and estrogen) [111]. Further, the hormonal dysregulation which is affected by nutritional lifestyle in this population is linked to insulin resistance, resulting in AD via this pathway as well [111].

Moreover, according to the included studies [19,23], the progression of mild cognitive impairment (MCI) in AD is of great interest, especially in patients with T2DM and MCI, because the early identification of these patients is the key for prevention of severe cognitive decline. Early remodeling of brain structural networks in these patients has been detected by diffusion tensor imaging (DTI) [112].

The metabolic risk in AD is further supported by findings indicating defects in the insulin signaling pathway irrespective of diabetes [113]. This phenomenon suggests that brain insulin resistance in AD patients could be an adverse outcome of Ab aggregation independently of peripheral insulin resistance or diabetes [114]. Hence, a method to simulate brain insulin resistance in experimental animal models is important, and it has been proven that intracerebroventricular injection of streptozotocin in rats is a valid way to mimic brain insulin resistance and, by extension, many aspects of sporadic AD [115,116]. As already mentioned, sporadic AD is complex because of its multifactorial heterogeneity. In particular, there are studies in rats that try to incorporate as many as possible etiological and risk factors, including the consequences of neuroinflammation, amyloid deposition, tau hyperphosphorylation and even changes of the hypothalamic–pituitary–adrenal (HPA) axis in cognitive impairment. An important AD-related vascular factor is also chronic cerebral hypoperfusion (CCH), provoked experimentally in rats via bilateral carotid artery stenosis, which has been referred to in these studies and indicates another major exacerbating factor in sporadic AD spectrum, interacting with amyloid toxicity and/or brain insulin resistance [116].

Generalization of the findings on DM as a risk factor for AD should also be confirmed by future studies on populations with a different prevalence of DM and different ethnic backgrounds, including the Mediterranean countries.

## Figures and Tables

**Table 1:** Studies separated by different measures of probability.

	Study	Value (95% CI)	Number of Participants
RR	Becker et al. [17]	1.4 (0.7–3.1)	288
	Toro et al. [19]	1.18 (0.49–2.87)	381
	Cheng et al. [20]	1.5 (0.92–2.45)	1488
	Li et al. [23]	1.62 (1.00–2.62)	837
	Treiber et al. [33]	3.3 (2.5–4.3)	3634
OR	Tolppanen et al. [26]	1.25 (1.16–1.36)	28,093
	Giuseppe et al. [27]	0.98 (0.8–1.2)	6553
	Kadohara et al. [28]	1.31 (0.9–1.92)	1855
	Kim et al. [31]	1.77 (1.52–2.06)	84,144
HR	Irie et al. [16]	1.62 (0.98–2.67)	2547
	Raffaitin et al. [18]	1.15 (0.64–2.05)	7087
	Ohara et al. [21]	2.05 (1.18–3.57)	1017
	Kimm et al. [22]	1.6 (1.3–2.0)	848,505
	Wang et al. [24]	1.45 (1.38–1.52)	1,230,403
	Huang et al. [25]	1.76 (1.5–2.07)	142,744
	Yu et al. [30]	1.13 (1.11–1.15)	1,917,702
	Chung Li et al. [29]	1.32 (1.11–1.58)	16,706
	Longjian et al. [32]	1.22 (1.13–1.31)	63,117

## Data Availability

Not applicable.
